# Effect of a multivitamin preparation supplemented with phytosterol on serum lipids and infarct size in rats fed with normal and high cholesterol diet

**DOI:** 10.1186/1476-511X-12-138

**Published:** 2013-09-25

**Authors:** Tamás Csont, Márta Sárközy, Gergő Szűcs, Csilla Szűcs, Judit Bárkányi, Péter Bencsik, Renáta Gáspár, Imre Földesi, Csaba Csonka, Csaba Kónya, Péter Ferdinandy

**Affiliations:** 1 Cardiovascular Research Group, Department of Biochemistry, Faculty of Medicine, University of Szeged, Szeged, Hungary; 2 Pharmahungary Group, Szeged, Hungary; 3 Béres Pharmaceuticals Ltd, Budapest, Hungary; 4 Department of Laboratory Medicine, Faculty of Medicine, University of Szeged, Szeged, Hungary; 5 Department of Pharmacology and Pharmacotherapy, Faculty of Medicine, Semmelweis University, Budapest, Hungary

**Keywords:** Multivitamin, Multimineral, Prevention, Hypercholesterolemia, Cardiovascular risk, Inflammation, Oxidative stress

## Abstract

**Background:**

Although complex multivitamin products are widely used as dietary supplements to maintain health or as special medical food in certain diseases, the effects of these products were not investigated in hyperlipidemia which is a major risk factor for cardiovascular diseases. Therefore, here we investigated if a preparation developed for human use containing different vitamins, minerals and trace elements enriched with phytosterol (VMTP) affects the severity of experimental hyperlipidemia as well as myocardial ischemia/reperfusion injury.

**Methods:**

Male Wistar rats were fed a normal or cholesterol-enriched (2% cholesterol + 0.25% cholate) diet for 12 weeks to induce hyperlipidemia. From week 8, rats in both groups were fed with a VMTP preparation or placebo for 4 weeks. Serum triglyceride and cholesterol levels were measured at week 0, 8 and 12. At week 12, hearts were isolated, perfused according to Langendorff and subjected to a 30-min coronary occlusion followed by 120 min reperfusion to measure infarct size.

**Results:**

At week 8, cholesterol-fed rats showed significantly higher serum cholesterol level as compared to normal animals, however, serum triglyceride level did not change. VMTP treatment significantly decreased serum cholesterol level in the hyperlipidemic group by week 12 without affecting triglyceride levels. However, VMTP did not show beneficial effect on infarct size. The inflammatory marker hs-CRP and the antioxidant uric acid were also not significantly different.

**Conclusions:**

This is the first demonstration that treatment of hyperlipidemic subjects with a VMTP preparation reduces serum cholesterol, the major risk factor for cardiovascular disease; however, it does not provide cardioprotection.

## Background

Large clinical studies showed that a significant population of adults is affected by hyperlipidaemia in the developed countries [1]. In the USA, approximately 100 million people (44.4%) suffered from hypercholesterolemia (>200 mg/dL) in 2008 [2]. It is well known, that hyperlipidemia, especially hypercholesterolemia is a major risk factor in the development of atherosclerosis and subsequent ischemic heart disease [3] which is a leading cause of death in industrialized countries [4]. Moreover, several experimental studies have demonstrated that in addition to its well-known pro-atherogenic effect in the vasculature, hyperlipidemia may directly affect the heart causing contractile dysfunction [5,6] and attenuated responses to cardioprotective interventions [7-10].

It has been shown in large clinical trials that antihyperlipidemic agents e.g. statins [11], fibrates [12], and niacin [13] could reduce the incidence of cardiovascular events in hypercholesterolemic patients [11]. Therefore, development of anti-hyperlipidemic strategies is a crucial point in reducing the risk of coronary heart disease.

Regular consumption of multivitamin and multimineral supplements is common in developed countries [14] to maintain general health. In the United States, more than half of the adult population use dietary supplements [15] primarily in the form of multivitamins with or without minerals [16]. In 1998 a study reported that in Germany 18% of men and 25% of women were regular users of multivitamins among 18–79 years old adults [17]. Moreover, sales data show increasing consumption of these products both in the USA and Europe. The effect of these complex multivitamin preparations on hyperlipidemia and its consequences is, however, not well understood.

Not only total energy intake and macronutrients including carbohydrates, protein and fat, but also micronutrients including vitamins, minerals and trace elements may affect the severity of hyperlipidaemia. A few clinical and experimental studies have shown that some individual vitamins and vitamin-like substances e.g. coenzyme Q10 [18], B3 [19], and folate [20,21], minerals e.g. iron [22] and copper [23], and trace elements e.g. selenium [24] beneficially affect hyperlipidaemia and its complications. In these studies, effects of individual vitamins, minerals and trace elements or combination of two or three components were investigated on hyperlipidemia. Interestingly, additional food supplements prepared from plants including phytosterols appeared on the market as functional food ingredients. However, results of large clinical studies on the lipid-lowering effects of plant sterols and stanols are controversial [25,26], and it is not known if phytosterols may provide additional benefit in protection of the ischemic heart.

Dietary supplements containing multivitamins, minerals and trace elements enriched with phytosterol now are available on the market. Surprisingly, there is only very limited literature data available on the effects of such preparations developed for human use on hyperlipidemia and its consequences [27].

Therefore, here we aimed to investigate if a commercial VMTP preparation containing 17 different vitamins, coenzyme Q10, minerals, trace elements and phytosterol affects the progression of hyperlipidemia and the severity of myocardial ischemia/reperfusion injury in a diet-induced experimental model of hyperlipidemia in rats.

## Methods

This investigation conforms to the National Institutes of Health Guide for the Care and Use of Laboratory Animals (NIH Pub. No. 85–23, Revised 1996) and was approved by the Animal Research Ethics Committee of the University of Szeged.

Six weeks old male Wistar rats (170–200 g initial body weight) were used in the study. Animals were housed in pairs in individually ventilated cages (Sealsafe IVC system, Italy) and were maintained in a temperature-controlled room with a 12-h:12-h light/dark cycles throughout the study. Standard rat chow and tap water were supplied ad libitum.

### Preparation of vitamins, minerals, trace elements and phytosterol (VMTP)

The VMTP preparation (“Actival Szterin film-coated tablet”, Béres Pharmaceuticals, Budapest, Hungary; for content see Table 1 and Additional file 1: Table S1) investigated in the present study is a commercially available food supplement in several countries in Europe. The individual components and their daily doses of the VMTP preparation were selected by the manufacturer on the basis of their individual preclinical and clinical efficacy and safety data available in the literature taking into consideration the Nutritive Reference Values [28].

**Table 1:** Ingredients of the VMTP preparation

**Active ingredients**	**Amount of ingredient/1 g product**	Daily dose*
Phytosterol	377.4 mg	39.62 mg/kg/day
Coenzim Q10	5.66 mg	0.59 mg/kg/day
Vitamin D3	1.89 μg (75.6 IU)	0.20 μg/kg/day (8 IU/kg/day)
Vitamin B1 (Thiamine)	0.42 mg	0.04 mg/kg/day
Vitamin B2 (Riboflavin)	0.53 mg	0.06 mg/kg/day
Vitamin B3 (Nicotinic acid)	6.04 mg	0.63 mg/kg/day
Vitamin B6 (Pyridoxine)	0.53 mg	0.06 mg/kg/day
Vitamin B12 (Cyanocobalamine)	0.94 μg	0.10 μg/kg/day
Biotin	18.87 μg	1.98 μg/kg/day
Pantothenic acid	2.26 mg	0.24 mg/kg/day
Folic acid	75.47 μg	7.92 μg /kg/day
Iron	2.64 mg	0.28 mg/kg/day
Manganese	0.38 mg	0.04 mg/kg/day
Copper	0.19 mg	0.02 mg/kg/day
Selenium	6.92 mg	0.73 mg/kg/day
Zinc	1.89 mg	0.20 mg/kg/day
Iodine	28.30 μg	2.97 μg/kg/day

* To conform to the human daily dose of the preparation, rat daily dose was adjusted according to the ratio of human and rat body surface areas.

### Experimental protocol

**Figure 1:**
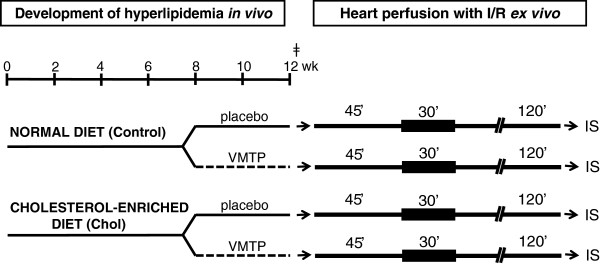
**Experimental protocol.** Male Wistar rats were fed with normal or 2% cholesterol and 0.25% cholic acid enriched diet to induce experimental hyperlipidemia. At week 8, fasting blood cholesterol and triglyceride measurement were performed in order to verify the development of hyperlipidemia. Both normolipidemic and hyperlipidemic rats were then fed with placebo or a mixture of vitamins, micro and trace elements (VMTP) supplemented with phytosterol for 4 weeks. At week 12, fasting blood cholesterol and triglyceride measurement were performed to monitor the effect of VMTP supplemented with phytosterol on hyperlipidemia. Hearts were then isolated, perfused according to Langendorff and subjected to a 30-min coronary occlusion followed by 120 min reperfusion to measure infarct size.

Male Wistar rats were fed a normal or a 2% cholesterol plus 0.25% cholic acid-enriched diet for 12 weeks to induce experimental hyperlipidemia (Figure 1). At week 0 and 8, fasting serum cholesterol as well as triglyceride measurements were performed in order to verify the development of hyperlipidemia (Figure 1). From week 8, both normolipidemic and hyperlipidemic rats were fed with a VMTP preparation (105 mg/kg/day) or placebo (57.5 mg/kg/day) for 4 weeks (Figure 1). To conform to the human daily dose of the preparation, rat daily dose was adjusted according to the ratio of human and rat body surface areas. Then fasting serum cholesterol and triglyceride measurements were performed at week 12 to monitor the effect of multivitamin treatment on hyperlipidemia (Figure 1). At week 12, rats were anaesthetized using diethyl ether. Hearts were isolated (Figure 1), and perfused according to Langendorff as described earlier [29]. The perfused hearts were then subjected to a 30-min regional ischemia and a 120-min reperfusion. At the end of the perfusion protocol, the coronary artery was reoccluded and the area at risk and the infarcted area were delineated using an Evans blue/triphenyltetrazolium chloride double staining method [30,31] (Figure 1).

### Measurement of serum cholesterol and triglyceride levels

To monitor the effect of 2% cholesterol plus 0.25% cholic acid-enriched diet as well as VMTP treatment on serum lipid levels, serum cholesterol and triglyceride levels were measured at week 0, 8 and 12 using a test kit supplied by Diagnosticum Zrt. (Budapest, Hungary) as described previously [6,32].

### Ex vivo cardiac perfusions and infarct size determination

At week 12, rats were anesthetized and hearts were isolated and perfused at 37°C according to Langendorff with oxygenated Krebs-Henseleit buffer as previously described [31,33,34]. Hearts were subjected to 45 minutes of aerobic perfusion followed by test ischemia-reperfusion induced by a 30-min occlusion of the left descending coronary artery. A 3–0 silk suture was placed around the origin of the left descending coronary artery and passed through a plastic tube to form a snare. After stabilization of the heart, coronary occlusion was induced by pulling the ends of the suture taut and clamping the snare onto the epicardial surface. Reperfusion was achieved by releasing the snare as previously described [31,34] (Figure 1). At the end of the 2-h reperfusion protocol, the coronary artery was reoccluded and 5 ml of 0.1% Evans blue dye (Merck, Germany) was injected into the aorta to delineate the area-at-risk zone. Stained hearts were weighed, frozen, sliced, and incubated at 37°C in 1% triphenyl-tetrazolium chloride (Sigma Aldrich, Germany) to delineate infarcted tissue. Slices were then fixed and quantified by planimetry using Infarctsize 2.5 software (Pharmahungary, Szeged, Hungary) [32]. Infarct size was expressed as a percentage of the area-at-risk zone [30]. The area at risk was calculated as a percentage of total ventricular area [30].

### Measurement of plasma hs-CRP and uric acid levels

Plasma hs-CRP level was measured as a systemic marker of inflammation at week 12 by a commercially available immunturbidimetric kit from Roche Diagnostics (Mannheim, Germany) according to the instructions of the manufacturer. The functional sensitivity of hs-CRP assay was 0.11 mg/L and the measuring range was between 0.1-20 mg/L.

Plasma uric acid level was measured as a general antioxidant marker at week 12 by a colorimetric kit provided by Roche Diagnostics (Mannheim, Germany) according to the instructions of the manufacturer. The detection limit of the assay was 11.9 μmol/L and the measuring range was between 11.9-1487 μmol/L.

### Statistical analysis

Statistical analysis was performed by using Sigmaplot 12.0 for Windows (Systat Software Inc). All values are presented as mean ± SEM. Two way ANOVA was used to determine the effect of hyperlipidemia or VMTP on fasting serum cholesterol and triglyceride levels, plasma hs-CRP and uric acid levels, as well as on the infarct size. P < 0.05 was accepted as a statistically significant difference.

## Results

### Effect of cholesterol-enriched diet on serum lipid levels

**Figure 2:**
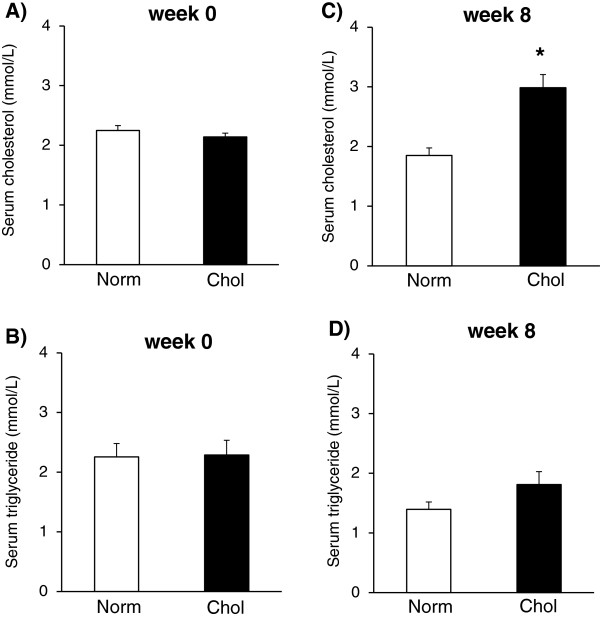
**Effect of 2% cholesterol plus 0.25% cholic acid-enriched diet on serum cholesterol and triglyceride levels.** Serum cholesterol level at week 0 **(Panel A)** and week 8 **(Panel C)** as well as serum triglyceride level at week 0 **(Panel B)** and week 8 **(Panel D)**. Values are means ± SEM, *p < 0.05 normal vs. cholesterol-enriched diet; n = 22 in each group.

In order to verify the development of hyperlipidemia in rats fed a 2% cholesterol plus 0.25% cholic acid-enriched diet, concentrations of fasting serum triglyceride and cholesterol levels were determined at week 0 and 8 (Figure 2). Baseline serum triglyceride and cholesterol levels did not differ between groups at week 0. Cholesterol-fed rats showed a significantly higher serum cholesterol level as compared to normal rats at week 8 confirming the development of hypercholesterolemia (Figure 2C). However, serum triglyceride level was not significantly affected by cholesterol diet at week 8 (Figure 2D).

### Effect of VMTP treatment on serum lipid levels, body weight and heart weight

**Figure 3:**
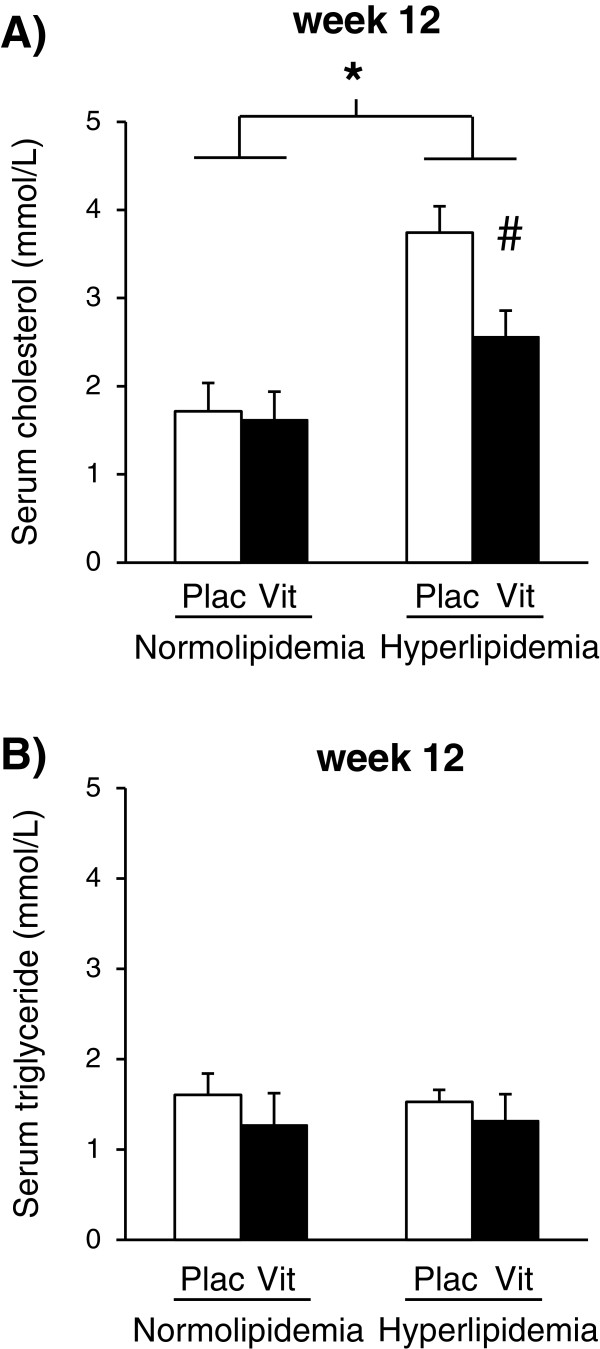
**Effect of the VMTP preparation on serum cholesterol and triglyceride levels.** Serum cholesterol level at week 12 **(Panel A)** and serum triglyceride level at week 12 **(Panel B)**. Values are means ± SEM, *p < 0.05 normal vs. cholesterol-enriched diet; # p < 0.05 placebo vs. VMTP treatment, n = 11 in each group.

In order to monitor the effect of VMTP on serum lipid levels, concentrations of fasting serum triglyceride and cholesterol were determined after 4 weeks of treatment (at week 12) in both normo- and hyperlipidemic groups (Figure 3). In normolipidemic animals, VMTP treatment did not affect serum triglyceride or cholesterol levels at week 12 (Figures 3A and 3B). In hyperlipidemic animals, VMTP treatment significantly decreased serum cholesterol level as compared to hyperlipidemic placebo-treated group at week 12 (Figure 3A), however, it did not change serum triglyceride level (Figure 3B). Body weight or heart weight was not significantly different among the experimental groups (Table 2).

**Table 2:** Effect of VMTP preparation on body weight, heart weight and coronary flow (CF)

**Parameter**	**Normolipidemia**		**Hyperlipidemia**		**Significance**
	**Placebo**	**VMTP**	**Placebo**	**VMTP**	
Body weight (g)	472 ± 56	461 ± 25	480 ± 58	480 ± 35	ns
Heart weight (g)	1.59 ± 0.75	1.49 ± 0.55	1.6 ± 0.76	1.66 ± 0.90	ns
(Heart weight/body weight)*1000	3.38 ± 0.12	3.23 ± 0.17	3.33 ± 0.10	3.38 ± 0.15	ns
CF (mL/min) – before ischemia	17.4 ± 1.3	18.9 ± 1.5	19.3 ± 2.6	19.1 ± 2.2	ns
CF (mL/min) – first minute of ischemia	15.7 ± 2.1	20.3 ± 2.3	17.4 ± 4.2	19.9 ± 3.4	ns
CF (mL/min) – during ischemia	19.7 ± 1.2	20.6 ± 1.8	20.5 ± 2.3	25.2 ± 4.0	ns
CF (mL/min) - end of reperfusion	22.0 ± 5.8	16.8 ± 2.0	14.7 ± 2.2	20.3 ± 2.4	ns

### Effect of VMTP treatment on area at risk, infarct size and coronary flow

**Figure 4:**
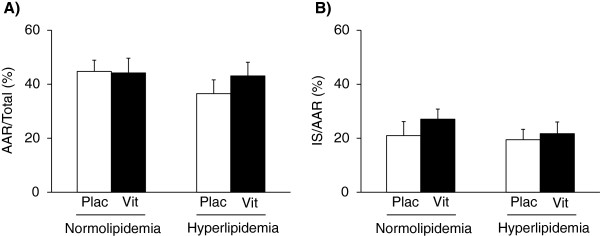
**Effect of the VMTP preparation on infarct size.** Area at risk **(Panel A)** and infarct size **(Panel B)** at week 12. Values are means ± SEM, n = 9-10.

Infarct size was measured at week 12 to investigate the severity of ischemia/reperfusion injury and the effect of VMTP treatment in normolipidemia as well as in hyperlipidemia. Neither the presence of hyperlipidemia nor the VMTP treatment had a significant effect on infarct size at week 12 (Figure 4). The area-at-risk zone and coronary flow were not affected significantly in any of the groups (Figure 4, Table 2).

### Effect of VMTP preparation on plasma hs-CRP and uric acid levels

Plasma hs-CRP level was measured as a systemic endogenous marker of inflammation. Neither the presence of hyperlipidemia nor the VMTP treatment had a significant effect on plasma hs-CRP level at week 12 (Table 3). Plasma level of uric acid, a well-known antioxidant, was not significantly different among the experimental groups (Table 3).

**Table 3:** Effect of VMTP preparation on plasma hs-CRP and uric acid levels

**Parameter**	**Normolipidemia**		**Hyperlipidemia**		**Significance**
	**Placebo**	**VMTP**	**Placebo**	**VMTP**	
hs-CRP (mg/L)	1.38 ± 0.14	1.19 ± 0.19	1.31 ± 0.12	1.65 ± 0.15	ns
Uric acid (μmol/L)	42.8 ± 12.5	64.8 ± 15.1	75.3 ± 15.3	57.6 ± 11.4	ns

## Discussion

In the present study we have shown that chronic treatment of hyperlipidemic adult male rats with a VMTP preparation containing 9 vitamins, coenzyme Q10, 5 micro-, and 1 trace element and phytosterol reduces serum cholesterol, the major risk factor for cardiovascular disease. However, this preparation failed to affect the severity of ischemia/reperfusion injury. This is the first demonstration that although VMTP preparation effectively reduces cholesterol level but does not provide cardioprotection.

Regular consumption of multivitamins supplemented with coenzyme Q10 and phytosterols for prevention or adjunctive treatment of cardiovascular risk factors is common in developed countries. According to our best knowledge, there are no preclinical studies available in the literature investigating the effect of a complex preparation of multivitamins, multiminerals, vitamin-like substances, and phytosterol on hyperlipidemia. Only one clinical pilot study evaluated the efficacy and safety of a similar complex preparation on serum lipid levels [27]. This study involving 25 children and adolescents with a borderline hypercholesterolemia (serum total cholesterol 180–240 mg/dL) has shown that a combination of plant sterol, fish oil and B vitamins significantly reduced serum total cholesterol, LDL- cholesterol, VLDL-cholesterol, subfractions LDL-2, IDL-1, IDL-2 and plasma homocysteine level after 16 weeks of treatment [27]. In our present study, we have shown that a commercially available VMTP preparation significantly reduced serum cholesterol level in hyperlipidemic but not in normolipidemic rats. However, it did not affect triglyceride levels in either normolipidemic or hyperlipidemic animals. These studies show that complex preparations of multivitamins and phytosterols beneficially affect the severity of hypercholesterolemia. In our present study, the VMTP preparation resulted in an approximately 25% reduction in serum cholesterol level in hyperlipidemic animals. However, clinical trials and their meta-analysis showed that phytosterol alone may result in an approximately 4-15% reduction in serum cholesterol level in hyperlipidemia [35]. Therefore, one may speculate that a combination of phytosterol with multivitamins, multiminerals, and coenzyme Q10 may have an additional benefit on lipid lowering, possibly via influencing unsaturated fatty acid levels or HMG-CoA activities. However, this assumption needs further preclinical and clinical studies.

It is well known that hyperlipidemia is a major risk factor of myocardial infarction and hyperlipidemia interferes with cardioprotective mechanisms [8]. However, interestingly there are no data available in the literature on the cardioprotective effect of any complex multivitamins. Therefore, here we investigated the effect of a VMTP preparation on myocardial infarct size and found that the VMTP preparation failed to affect infarct size in normal or cholesterol-fed animals. This is the first demonstration that a VMTP preparation although effectively reduced serum cholesterol levels it did not provide cardioprotection.

It should be mentioned that only one experimental study [36] supports the direct infarct size limiting effect of a phytosterol derivative in rats. However, there is a lack of gender distribution in the experimental population in this aforementioned study [36]. Taken together, our present study is the first demonstration that VMTP preparation effectively reduces cholesterol level but does not provide cardioprotection. Although the reason for the lack of cardioprotective effect by VMTP in our study is not known as no alterations were found in inflammatory markers and antioxidants, it seems that the VMTP preparation is not able to directly prevent necrosis in an acute model of myocardial infarction. However, it cannot be excluded that the VMTP preparation may be able to confer some cardioprotection in cases when infarct size is increased.

Our current study is limited in some aspects since it does not examine the mechanism of the effect of VMTP preparation and the individual contribution of the 17 different components and their intereactions in the present model of experimental hyperlipidemia and infarction. However, it needs to be emphasized that this particular preparation and others with similar compositions (multivitamins, vitamin-like substances e.g. coenzyme Q10, multiminerals, and phytosterols) are commercially available and regularly consumed by healthy population and that at risk of cardiovascular disease. Therefore, thorough investigations of the efficacy and safety of such products are important in cardiovascular risk. Future studies investigating the possible preventive effect of VMTP preparations on the development of hyperlipidemia are also needed.

## Conclusions

Although VMTP preparations are widely used in healthy population or by patients with cardiovascular risk factors including hyperlipidemia, our present study is the first preclinical demonstration that a VMTP preparation attenuates the progression of experimental hypercholesterolemia, however, it does not affect the severity of ischemia/reperfusion injury in the heart. Further preclinical and clinical studies are needed to optimize the compositions and to elucidate the efficacy, safety and the mechanism of the effect of widely used VMTP preparations.

## Competing interests

Béres Pharmaceuticals Ltd. was the leader of the consortial project funded by the National Development Agency (MED_FOOD TECH_08-A1-2008-0275).

## Author contributions

TC, CK and PF conception and design of research; MS, GS, RG, PB, CS, JB and IF performed experiments; MS, GS, PB, RG, CS, JB and CC analysed data; MS, GS, JB, IF and TC interpreted results of experiments; MS prepared figures; TC, and MS drafted manuscript; MS, TC and PF edited and revised manuscript; TC, MS, GS, CS, JB, PB, RG, CC, IF, CK and PF approved final version of manuscript.

## Supplementary Material

Additional file 1: Table S1 Ingredients of the Placebo. Click here for file
