# Supplementary material for: Effect of a multivitamin preparation supplemented with phytosterol on serum lipids and infarct size in rats fed with normal and high cholesterol diet
Source: Lipids Health Dis. 2013 Sep 25;12:138. doi: 10.1186/1476-511X-12-138 (PMC3851526; doi:10.1186/1476-511X-12-138)
Supplement: Additional file 1: Table S1 — Ingredients of the Placebo. [file 1476-511X-12-138-S1.docx]

**Additional file 1.Table S1:** Ingredients of the Placebo

| **Active ingredients** | **Amount of ingredient/**  **1 g product (mg/g)** | **Daily dose**  **(mg/kg/day)** |
| --- | --- | --- |
| Prosolv HD 90 | 188.68 | 19.81 |
| Starch 1500 | 15.09 | 1.58 |
| Pearlitol SD 200 | 228.99 | 24.04 |
| Disolcel GF | 30.19 | 3.17 |
| Aerosil 200 | 11.32 | 1.19 |
| Talcum | 18.87 | 1.98 |
| Magnesium stearate | 15.09 | 1.58 |
| Stearic acid | 9.06 | 0.95 |
